# Association Between Physical Activity and Risk of Depression: A Systematic Review and Meta-analysis

**DOI:** 10.1001/jamapsychiatry.2022.0609

**Published:** 2022-04-13

**Authors:** Matthew Pearce, Leandro Garcia, Ali Abbas, Tessa Strain, Felipe Barreto Schuch, Rajna Golubic, Paul Kelly, Saad Khan, Mrudula Utukuri, Yvonne Laird, Alexander Mok, Andrea Smith, Marko Tainio, Søren Brage, James Woodcock

**Affiliations:** 1 MRC Epidemiology Unit, University of Cambridge, Cambridge, England; 2 Centre for Public Health, Institute of Clinical Sciences, Queen’s University Belfast, Belfast, Northern Ireland; 3 Department of Sports Methods and Techniques, Federal University of Santa Maria, Santa Maria, Brazil; 4 Cambridge University Hospitals NHS Foundation Trust, Cambridge, England; 5 Physical Activity for Health Research Centre, Institute of Sport Physical Education and Health Science, University of Edinburgh, Edinburgh, Scotland; 6 University of Cambridge School of Clinical Medicine, Addenbrooke’s Treatment Centre, Cambridge Biomedical Campus, Cambridge, England; 7 Prevention Research Collaboration, Sydney School of Public Health, Faculty of Medicine and Health, The University of Sydney, Sydney, New South Wales, Australia; 8 Charles Perkins Centre, The University of Sydney, Sydney, New South Wales, Australia; 9 Singapore Institute for Clinical Sciences, Agency for Science, Technology and Research, Singapore; 10 Sustainable Urban Programme, The Finnish Environment Institute, Helsinki, Finland; 11 Systems Research Institute, Polish Academy of Sciences, Warsaw, Poland

## Abstract

**Importance:**

Depression is the leading cause of mental health–related disease burden and may be reduced by physical activity, but the dose-response relationship between activity and depression is uncertain.

**Objective:**

To systematically review and meta-analyze the dose-response association between physical activity and incident depression from published prospective studies of adults.

**Data Sources:**

PubMed, SCOPUS, Web of Science, PsycINFO, and the reference lists of systematic reviews retrieved by a systematic search up to December 11, 2020, with no language limits. The date of the search was November 12, 2020.

**Study Selection:**

We included prospective cohort studies reporting physical activity at 3 or more exposure levels and risk estimates for depression with 3000 or more adults and 3 years or longer of follow-up.

**Data Extraction and Synthesis:**

Data extraction was completed independently by 2 extractors and cross-checked for errors. A 2-stage random-effects dose-response meta-analysis was used to synthesize data. Study-specific associations were estimated using generalized least-squares regression and the pooled association was estimated by combining the study-specific coefficients using restricted maximum likelihood.

**Main Outcomes and Measures:**

The outcome of interest was depression, including (1) presence of major depressive disorder indicated by self-report of physician diagnosis, registry data, or diagnostic interviews and (2) elevated depressive symptoms established using validated cutoffs for a depressive screening instrument.

**Results:**

Fifteen studies comprising 191 130 participants and 2 110 588 person-years were included. An inverse curvilinear dose-response association between physical activity and depression was observed, with steeper association gradients at lower activity volumes; heterogeneity was large and significant (*I*^2^ = 74%; *P* < .001). Relative to adults not reporting any activity, those accumulating half the recommended volume of physical activity (4.4 marginal metabolic equivalent task hours per week [mMET-h/wk]) had 18% (95% CI, 13%-23%) lower risk of depression. Adults accumulating the recommended volume of 8.8 mMET hours per week had 25% (95% CI, 18%-32%) lower risk with diminishing potential benefits and higher uncertainty observed beyond that exposure level. There were diminishing additional potential benefits and greater uncertainty at higher volumes of physical activity. Based on an estimate of exposure prevalences among included cohorts, if less active adults had achieved the current physical activity recommendations, 11.5% (95% CI, 7.7%-15.4%) of depression cases could have been prevented.

**Conclusions and Relevance:**

This systematic review and meta-analysis of associations between physical activity and depression suggests significant mental health benefits from being physically active, even at levels below the public health recommendations. Health practitioners should therefore encourage any increase in physical activity to improve mental health.

## Key Points

**Question:**

What is the dose-response association between physical activity and incident depression in adults?

**Findings:**

This systematic review and meta-analysis of 15 prospective studies including more than 2 million person-years showed an inverse curvilinear association between physical activity and incident depression, with greater differences in risk at lower exposure levels. Adults meeting physical activity recommendations (equivalent to 2.5 h/wk of brisk walking) had lower risk of depression, compared with adults reporting no physical activity.

**Meaning:**

In this study, relatively small doses of physical activity were associated with substantially lower risks of depression.

## Teaser

This systematic review and meta-analysis examines data from published prospective studies of adults to gauge the dose-response association between physical activity and incident depression.

## Introduction

Depression is the leading cause of mental health–related disease burden and a major cause of disability worldwide, affecting approximately 280 million people and accounting for more than 47 million disability-adjusted life-years in 2019.^1^ Depression is also associated with premature mortality from other illnesses^2^ and suicide.^3^

Prevention of depression requires effective interventions, including modification of established risk factors.^4^ Narrative reviews have concluded that physical activity can prevent future depression.^5,6^ One meta-analysis of prospective studies reported that compared with people with low levels of physical activity, those with higher levels had 17% (95% CI, 12%-21%) lower odds of developing depression,^7^ while another meta-analysis reported 21% (95% CI, 18%-25%) lower odds when synthesizing 106 associations from 65 studies using diverse exposure definitions.^8^ To our knowledge, no study has yet synthesized the evidence to describe the strength or shape of the association by conducting a dose-response meta-analysis using harmonized exposure estimates.

Estimating the dose-response relationship between physical activity and any health outcome using meta-analysis is challenging because of the diversity of assessment and inconsistent reporting. Most often, the harmonization of different estimates of physical activity exposure is achieved using binary categorization of low vs high activity, but this approach results in loss of information and does not tell us about the variation in risk across a range of physical activity doses. In contrast, previous work has shown that by using more detailed exposure harmonization, it is possible to investigate the shape of the association between physical activity and type 2 diabetes using dose-response meta-analytical techniques.^9^ Examining the association between the dose of physical activity and depression in this way allows approximation of the reduction in risk associated with different levels of activity. By combining estimates of reduction in risk with population prevalence estimates of activity, it is possible to quantify the population burden of depression related to insufficient physical activity and the potential public health impact of activity-related interventions.

The aim of this systematic review and meta-analysis was to investigate the dose-response association between physical activity and depression. We also assessed the potential population changes in depression that may be preventable by higher physical activity levels.

## Methods

The study protocol is available at PROSPERO (CRD42018095507) and followed Meta-analysis of Observational Studies in Epidemiology (MOOSE) reporting guidelines.

### Eligibility Criteria

We included prospective cohort studies of adults (≥18 years of age) that reported any dimension of physical activity at 3 or more exposure levels and reported risk estimates for depression. Studies were excluded if the sample size was fewer than 3000 participants (to limit small-study effects indicative of publication bias) or if the follow-up period was less than 3 years (to minimize reverse causality bias from undiagnosed depression at baseline). Full details are provided in eTable 1 in the Supplement. Studies were eligible if the exposure included leisure-time physical activity, either alone or combined with other activity domains. The outcome of interest was depression, including (1) presence of major depressive disorder indicated by self-report of physician diagnosis, registry data, or diagnostic interviews using *DSM* criteria or *International Statistical Classification of Diseases and Related Health Problems, Tenth Revision, *codes F32 through F33; (2) elevated depressive symptoms established using validated cutoffs for a depressive screening instrument, eg, Center for Epidemiologic Studies Depression scale.

### Search and Selection

We searched PubMed, SCOPUS, Web of Science, PsycINFO, and the reference lists of systematic reviews and articles retrieved from the search or known to the authors. Specific search strings are available in eMethods 1 in the Supplement. We considered peer-reviewed articles published in academic journals until November 12, 2020. No language limits were set. Titles and abstracts and subsequently full-text articles were screened by 2 independent reviewers for eligibility, with disagreements resolved by a third reviewer (eFigure 1 in the Supplement).

### Data Extraction

Data extraction was carried out using a standardized extraction form and conducted independently by 2 researchers, with disagreements resolved by a third researcher. For each exposure category, we extracted information to estimate physical activity volume if not reported directly, number of depression cases, number of participants and person-years of follow-up, and the effect estimate with 95% CIs. Effect estimates from the most adjusted model were used. Results reported separately for men and women, or for multiple cohorts within a study, were extracted as separate associations.

When the data required for exposure harmonization or meta-analysis were not reported in the original articles, nor obtainable through other publications from the same cohort, we contacted authors before imputing critical information if necessary (eMethods 2 in the Supplement).

### Physical Activity Exposure Harmonization

We harmonized reported exposure levels to a common metric of physical activity volume in marginal metabolic equivalent task hours per week (mMET-h/wk), reflecting the energy expended above the resting metabolic rate (1 MET). Combinations of harmonization techniques were used depending on what was reported or obtained from authors and availability of validation work (details of the original exposure data, harmonization methods, and harmonization flowchart are available in an Open Science Framework^10^ repository).

#### Frequency, Duration, and Intensity Assumptions

Studies that described physical activity exposure as frequency and duration were converted to a measure of weekly duration. If session duration was not provided, a session duration of 0.75 hours was assumed. Weekly durations were then converted to activity volume in mMET hours per week by multiplying by intensity values of 1.5 mMET for light, 3.5 mMET for moderate and moderate to vigorous, and 7.0 mMET for vigorous physical activity.^11^ A score of 3.5 mMET corresponds to the midpoint of the range for moderate-intensity activity (≥2 to <5 mMETs). Vigorous was scored at 7 mMET because activity of this intensity contributes twice as much toward meeting physical activity guidelines compared with moderate activity (the World Health Organization advises 150-300 minutes of moderate intensity activity or 75-150 minutes of vigorous intensity activity per week^12^). Light activities are those in the range of 0.5 to 2 mMETs; however, these were scored slightly higher than the midpoint at 1.5 mMET to reflect the intensity of behaviors commonly referred to as light activity in questionnaires (eg, light housework, light gardening).

#### Converting Energy Expenditure to MET Units

One study reported energy expenditure without adjustment for body weight (kcal/wk). Weight was calculated using body mass index and height from national survey data.^13^ The exposure in kcal per week was then divided by derived weight and further converted to MET hours using 1 kcal/kg = 1 MET hour.

#### Removing the Resting Energy Expenditure Component of Physical Activity

For studies reporting physical activity volume and corresponding duration data, 1 MET hour per week was subtracted for each hour of activity undertaken; this is equivalent to the original studies having multiplied activity duration with net (mMET) rather than gross (MET) intensity values. If duration for each volume level was not reported or obtainable from authors, we used a prediction equation derived from studies where both volume and duration were available (eMethods 3 in the Supplement).

#### Removing Occupational Component of Aggregate Physical Activity Estimates

Nonoccupational physical activity was used as the exposure in all but 1 study^14^ that used total physical activity, including leisure-time, domestic, occupational, and transport domains. Given no domain-specific activity results were available for this study, we assumed occupational activity to be 40 hours per week at 1.25 METs (or 0.25 mMETs, ie, equivalent to sedentary office work) and estimated nonoccupational physical activity by subtracting this assumed occupational volume.

### Analyses

We conducted dose-response meta-analyses for outcomes with at least 4 independent studies available. For studies reporting only sex-stratified results, strata-specific risks were combined using fixed-effect meta-analysis. Sex-stratified meta-analyses were not conducted because only 2 sets of sex-stratified results were available.

A 2-stage random-effects meta-analysis was used to model the dose-response association between physical activity and depression. First, study-specific associations were estimated using generalized least-squares regression.^15,16^ Second, we estimated the pooled association by combining the study-specific coefficients using restricted maximum likelihood.^17^ We assumed the dose-response associations were nonlinear and modeled them by fitting restricted cubic splines with 3 knots at the 0, 37.5th, and 75th percentiles of person-years. If the statistical model was unable to converge for these knots, we increased the percentile for the upper knot until it did.

#### Estimated Population Risk

To provide a population perspective of the relative importance of the estimated dose-response associations, potential impact fractions (PIFs) were calculated based on the exposure prevalence in the populations of included cohorts.^18^ PIFs were calculated for 3 exposure levels based on the World Health Organization recommendations for adults^12^: the minimum recommended level of 8.8 mMET hours per week (volume equivalent to approximately 2.5 h/wk of physical activity at moderate intensity of 3.5 marginal METs), the level recommended for additional health benefits of 17.5 mMET hours per week, as well as 4.4 mMET hours per week (half the minimum recommended level).

#### Sensitivity and Subgroup Analysis

We conducted 2 sensitivity analyses. First, we assigned 2.5 and 6 mMET to moderate and vigorous physical activity, respectively, when intensity had to be assumed for physical activity exposure harmonization; for studies requiring assumptions about duration of sessions, we assigned a 0.5-hour duration (rather than 0.75-hour duration). Second, we tested the use of knots at the 0, 42.5th, and 85th percentiles in the cubic spline functions.

We conducted subgroup analyses by depression diagnosis method using 2 outcome subtypes: (1) major depression from registry data, self-report of physician diagnosis, or diagnostic interview; (2) use of a screening tool for elevated depressive symptoms with validated cutoffs.

#### Analysis of Heterogeneity

Cochran *Q* test and *I^2^* statistic were used to assess heterogeneity of effect measures between studies. We also conducted random-effect meta-regressions (at the physical activity level of 8.8 mMET-h/wk) using restricted maximum likelihood to identify how much of the variance in effect measures was due to proportion of men and women (women only, men only, mixed), outcome ascertainment (registry data, self-report of physician diagnosis, diagnostic interview, Center for Epidemiologic Studies Depression scale, other depressive symptoms scale), duration of follow-up (above or below median of 8.5 years), exclusion of prevalent cases at baseline (yes, no), handling other morbidities at baseline (exclusion of participants, statistical adjustment), and our exposure harmonization approaches (measurement unit conversion, measurement unit conversion plus occupational physical activity assumption, duration/intensity/frequency assumptions). See eTable 2 in the Supplement for more details. We conducted a leave-one-out sensitivity analysis to investigate the influence of each study on the overall effect-size (at the physical activity level of 8.8 mMET-h/wk).

#### Software, Data, and Code Availability

Analyses were performed using R, version 4.0.5, and the dosresmeta package,^19^ version 2.0.1. An interactive interface to visualize dose-response curves was developed using the Shiny package, version 1.0.5. Codes for all analyses and the interactive interface are available in a repository on GitHub^20^ (see README file).

## Results

### Literature Search

The systematic literature search yielded 19 175 results following removal of duplicates. After excluding 18 827 records based on title and abstract screening, 348 full-text articles were reviewed. Full-text screening identified 15 eligible publications reporting 15 associations including 191 130 participants contributing 28 806 incident depression events and 2 110 588 person-years. Approximately 64% of participants in the studies were women. All but 1 of the included studies originated in high-income countries: 6 from the United States,^21,22,23,24,25,26^ 6 from Europe,^27,28,29,30,31,32^ 1 from Australia,^33^ 1 from Japan,^34^ and 1 study that included data from India, Ghana, Mexico, and Russia.^14^ Study characteristics are described in Table 1.^14,21,22,23,24,25,26,27,28,29,30,31,32,33,34,35,36,37,38,39,40,41,42,43^

**Table 1.** Study Characteristics and Exposure Harmonization

Source (country)	Study name	Participants (cases), No.^a^	Sex	Age range at baseline, y	Follow-up time, y	Outcome subtype	Outcome ascertainment	Exposure harmonization^b^
Mikkelsen et al,^27^ 2010 (Denmark)	Copenhagen City Heart Study	8804 (436)	Women	20-93	≤26	Major depression	Registry	Used duration category midpoints. Multiplied by intensity (mMET) for light (1.5) and moderate (3.5) activity.
Paffenbarger et al,^24^ 1994 (United States)	Harvard Alumni Health Study	10 201 (387)	Men	35-74	23-27	Major depression	Self-report of physician diagnosis	Interpolated to get median BMI for sample. Mean height for men aged 35-74 y, 173 cm. Used these data to get weight by category. Divided midpoint of absolute activity volume by weight to give kcal/kg/wk. which is MET-h/wk. Equation used to remove resting metabolic rate component to give mMET-h/wk.
Ten Have et al,^32^ 2011 (the Netherlands)	Netherlands Mental Health Survey and Incidence Study	3998 (244)	Both (49% women)	18-64	3	Major depression	Electronic CIDI^35^	Used duration category midpoints. Multiplied by intensity (mMET) for moderate (3.5) activity.
Chang et al,^25^ 2016 (United States)	Nurses’ Health Study	21 728 (3945)	Women	≥65	≤10	Major depression	Self-report of depressive symptoms, use of antidepressants, physician diagnosis	Used duration category midpoints. Multiplied by intensity (mMET) for moderate (3.5) activity.
Cabello et al,^14^ 2017 (Ghana, India, Mexico, Russia)	World Health Organization’s Study on Global Aging and Adult Health	5970 (594)	Both (67% women)	≥18	3-8	Major depression	World Mental Health Survey Initiative version of the CIDI,^36^ self-report of physician diagnosis, medication, or treatment	Assigned MET-h/wk based on scoring described and used equation to remove resting metabolic rate component. Instrument does not capture sedentary behaviors, so no volume removed for assumed occupational physical activity.
Fernandez-Montero et al,^31^ 2020 (Spain)	Seguimiento Universidad de Navarra	15 488 (870)	Both (60% women)	18-64	Mean (SD): 8.5 (4.4)	Major depression	Self-report of physician diagnosis	Used volume category midpoints. Equation used to remove resting metabolic rate component to give mMET-h/wk.
Hallgren et al,^29^ 2019 (Sweden)	Swedish National March Cohort Study	20 594 (289)	Both (65% women)	≥18	≤13	Major depression	Registry	Used duration category midpoints. Multiplied by intensity (mMET) for moderate (3.5) activity.
España-Romero et al,^21^ 2013 (United States)	Aerobics Center Longitudinal Study	5110 (641)	Both (20% women)	20-83	Mean (range): 6.1 (1-12)	Elevated depressive symptoms	10-Item CES-D scale^37^	Used volume category midpoints. Equation used to remove resting metabolic rate component to give mMET-h/wk.
Camacho et al,^23^ 1991 (United States)	Alameda County Study	3664 (733)	Men	≥20	9	Elevated depressive symptoms	Human Population Laboratory depression index^38^	Article reported activity index 0-14 based on frequency of 4 behaviors; 14 represents often engaging in sports, swimming or walking, doing exercises, and gardening. The mMET-h/wk for a score of 14 was calculated by assuming frequency and duration and by assigning mMET based on description of activities. Midpoints of the index ranges of the 3 categories (0-4 [2], 5-8 [6.5], 9-14 [11.5]) were taken and assigned mMET-h/wk relative to the known score of an index of 14.
Pavey et al,^33^ 2013 (Australia)	Australian Longitudinal Study on Women’s Health	12 094 (2419)	Women	45-53	3	Elevated depressive symptoms	10-Item CES-D scale^37^	Used volume category midpoints. Equation used to remove resting metabolic rate component to give mMET-h/wk.
Wise et al,^22^ 2006 (United States)	Black Women’s Health Study	35 224 (9465)	Women	21-69	3-5	Elevated depressive symptoms	20-Item CES-D scale^39^	Used duration category midpoints. Multiplied by intensity (mMET) for vigorous (7) activity.
Hamer et al,^28^ 2009 (England)	English Longitudinal Study of Aging	4323 (348)	Both (53% women)	≥50	4	Elevated depressive symptoms	8-Item CES-D scale^40^	Used frequency category midpoints. Assumed session duration of 0.75 h. Multiplied by intensity (mMET) for light (1.5), moderate (3.5), and vigorous (7) activity.
Kuwahara et al,^34^ 2015 (Japan)	Japan Epidemiology Collaboration on Occupational Health Study	29 082 (6177)	Both (15% women)	20-64	Mean: 4.7	Elevated depressive symptoms	13-Item cohort specific scale^41^	Used volume category midpoints. Equation used to remove resting metabolic rate component to give mMET-h/wk.
Harvey et al,^30^ 2018 (Norway)	Health Study of Nord-Trøndelag County	22 564 (1578)	Both (50% women)	≥20	9-13	Elevated depressive symptoms	14-Item Hospital Anxiety and Depression scale^42^	Used duration category midpoints. Multiplied by intensity (mMET) for moderate (3.5) activity.
Hughes et al,^26^ 2019 (United States)	Health Professionals Follow-up Study	6311 (788)	Men	40-75	25	Elevated depressive symptoms	5-Item Mental Health Inventory^43^	Used volume category means. Equation used to remove resting metabolic rate component to give mMET-h/wk.

Abbreviations: BMI, body mass index; CES-D, Center for Epidemiologic Studies Depression scale; CIDI, Composite International Diagnostic Interview; mMET, marginal metabolic equivalent of task.

^a^ Cases of incident depression.

^b^ Volume category refers to the description of physical activity volume in the article, eg, <10, 10-20, and >20 MET-h/wk. Duration category refers to the description of physical activity duration in the article, eg, 0, 1-3, and >3 h/wk. In both cases, these ranges had to be converted to point estimates for the meta-analysis (further details available in Open Science Framework^10^ repository).

### Dose-Response Analyses

**Figure 1.**
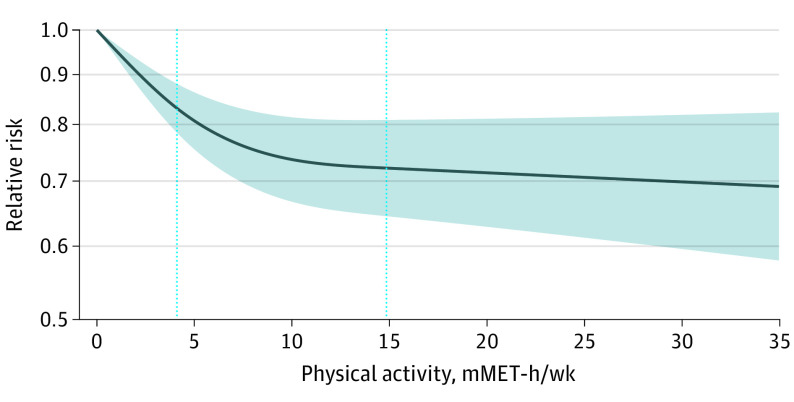
Association Between Physical Activity and Incidence of Depression Dark line represents the meta-analytical dose-response curve (constrained to be linear beyond upper knot at 75% of person-years). Shaded area displays 95% CI. Vertical dotted lines indicate knots at the 37.5th and 75th percentiles of person-years. *I*^2^ = 73.7%; *P* < .001. Interactive dose-response curves and exposure distributions are available online.^44^

**Figure 2.**
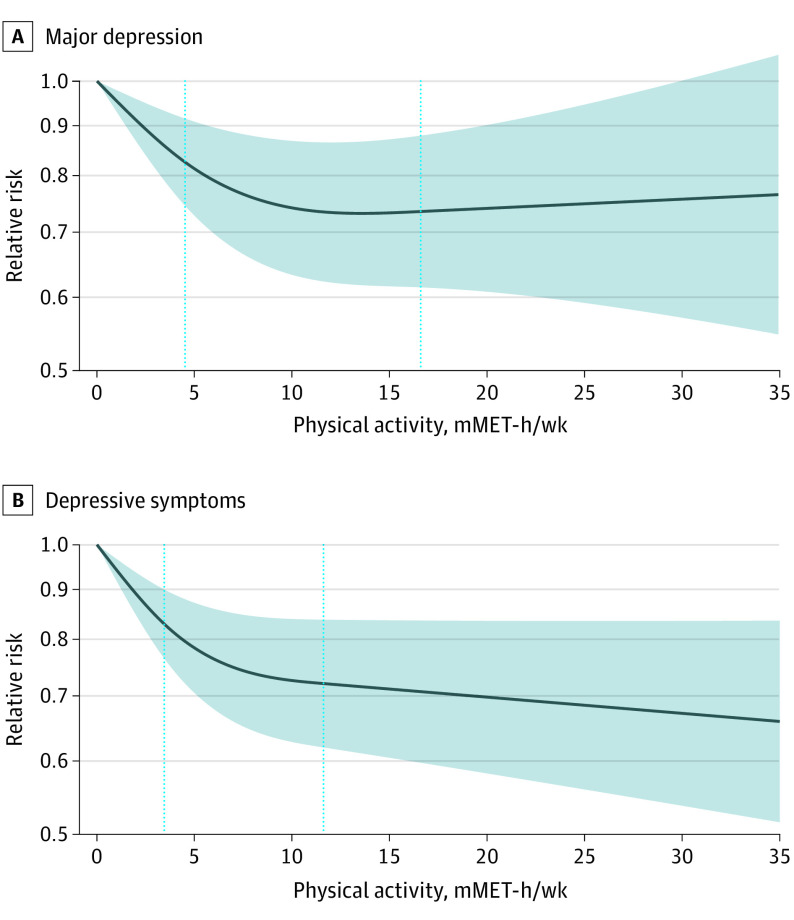
Associations Between Physical Activity and Incidence of Major Depression and Elevated Depressive Symptoms Dark lines represent the meta-analytical dose-response curve (constrained to be linear beyond upper knot at 75% of person-years). Shaded area displays 95% CIs. Vertical dotted lines indicate knots at the 37.5th and 75th percentiles of person-years. A, Major depression *I*^2^ = 54.2%; *P* = .01. B, Elevated depressive symptoms *I*^2^ = 81.3%; *P* < .001.

The majority (78%) of participants reported exposure levels below 17.5 mMET hours per week, with almost all (95%) data below 35 mMET hours per week; participants in studies of elevated depressive symptoms tended to be less active than those in studies of major depression (eFigure 2 in the Supplement). Figure 1 shows an inverse, curvilinear dose-response association between physical activity and depression, with greater differences in risk in the lower-dose region. Relative to adults not reporting any activity, those accumulating half the recommended volume of physical activity (4.4 mMET-h/wk) had 18% (95% CI, 13%-23%) lower risk of depression. Adults accumulating the recommended volume of 8.8 mMET hours per week had 25% (95% CI, 18%-32%) lower risk with diminishing potential benefits and higher uncertainty observed beyond that exposure level (Table 2). Interactive dose-response curves and exposure distributions are available online.^44^ The dose-response curves for major depression and elevated depressive symptoms showed similar curvilinear relationships (Table 2 and Figure 2).

**Table 2.** Relative Risk and Potential Impact Fractions of Incident Depression, Major Depression, and Elevated Depressive Symptoms at 3 Physical Activity Levels^a^

	Risk association by activity volume [mMET-h/wk], RR (95% CI)			Population impact by activity volume [mMET-h/wk], PIF (95% CI), %		
	4.4	8.8	17.5	4.4	8.8	17.5
Depression	0.82 (0.77-0.87)	0.75 (0.68-0.82)	0.72 (0.64-0.81)	6.38 (4.25-8.63)	11.53 (7.69-15.43)	13.89 (8.44-19.25)
Major depression	0.83 (0.75-0.92)	0.75 (0.64-0.87)	0.74 (0.61-0.88)	2.97 (1.27-4.91)	7.28 (3.36-11.44)	8.04 (2.38-13.82)
Elevated depressive symptoms	0.80 (0.73-0.88)	0.73 (0.64-0.84)	0.70 (0.59-0.84)	9.45 (5.19-13.86)	14.44 (7.88-20.92)	17.01 (8.39-25.24)

Abbreviations: mMET, marginal metabolic equivalent of task; PIF, potential impact fraction; RR, relative risk.

^a^ Risks are expressed relative to accumulating 0 mMET-h/wk.

### Estimated Population Risk

Based on PIF analyses of prevalences of physical activity and depression outcomes in the included cohorts, 11.5% (95% CI, 7.7%-15.4%) of incident depression could have been prevented if all adults had achieved at least 8.8 mMET hours per week of physical activity (Table 2). PIFs were approximately twice as high for elevated depressive symptoms than for major depression at both 8.8 and 17.5 mMET hours per week and approximately 3 times higher at 4.4 mMET hours per week (Table 2).

### Sensitivity Analyses

Using alternative assumptions of 0.5 hours for session duration and 1 MET lower-intensity values for moderate and vigorous physical activity (only when those assumptions were necessary) or alternative placement of knots for the splines did not materially alter dose-response associations (eTable 3 in the Supplement) or PIFs (eTable 4 in the Supplement).

### Analysis of Heterogeneity

Differences between proportion of women, exposure harmonization method, outcome ascertainment, duration of follow-up, exclusion of prevalent cases at baseline, or managing other morbidities at baseline did not significantly explain variance in the effect measures of associations between physical activity and depression (eFigure 3 in the Supplement). The leave-one-out sensitivity analysis did not identify any outliers (eFigure 4 in the Supplement).

## Discussion

This study reports the first dose-response meta-analysis of associations between physical activity and incident depression to our knowledge. Our results show an inverse curvilinear association with the greatest differences in risk observed between low doses of physical activity, suggesting most benefits are realized when moving from no activity to at least some. Accumulating an activity volume equivalent to 2.5 hours of brisk walking per week was associated with 25% lower risk of depression, and at half that dose, risk was 18% lower compared with no activity. Only minor additional benefits were observed at higher activity levels.

Previous meta-analyses have shown lower risks of depression among adults reporting high vs low physical activity^7^ and suggested a dose-response association using meta-regression without quantifying the association.^8^ Our study directly models how risk of depression varies across the physical activity exposure range using a continuous scale rather than cruder categories. We also found that even small volumes of activity were beneficial but go further by quantifying differences in risk for these doses. Our findings therefore have important new implications for health practitioners making lifestyle recommendations, especially to inactive individuals who may perceive the current recommended target as unrealistic.

The associations we observed are likely explained by more than 1 mechanism. Proposed pathways include acute neuroendocrine and inflammatory responses to activity such as activation of the endocannabinoid system (“runner’s high”)^45^ and longer-term adaptations, including changes in the brain’s neural architecture.^46^ Psychosocial and behavioral explanations have also been suggested, including improved physical self-perceptions and body image, more social interactions, and the personal development of coping strategies.^46^ The social aspect of activity participation may operate even at relatively low doses, consistent with the dose-response curve we observed. The role of the environment as a potential moderator of the association between physical activity and depression should also be considered. For example, the use of green space is associated with lower risk of depression,^47^ with mediation analysis suggesting only part of the association is explained by physical activity.^48^ Conversely, noise pollution^49^ and neighborhood deprivation^50^ might diminish the mental health benefits of activity. Such contextual factors may have contributed to the high level of heterogeneity we observed between studies. The above mechanisms may not operate to the same degree across different types, frequencies, intensities, and contexts of activity,^51^ and measurement of these activity subdimensions may also differ between studies, potentially resulting in higher heterogeneity. Future work should therefore explore the shape of the dose-response relationship for these aspects of activity in addition to total volume.

In subgroup analyses using results from different cohorts for each outcome, we observed that the dose response with physical activity was similar for major depression and elevated depressive symptoms, as has been reported using binary exposure expressions.^7^ However, PIFs for elevated depressive symptoms were higher; this is because participants in those studies were generally less active than participants in the studies examining major depression. Examining depressive symptoms on a continuous scale instead of using dichotomized outcome variables may provide further insight regarding the benefits of physical activity to alleviate depressive symptoms, but no study that we reviewed reported results in this way.

To conduct nonlinear dose-response analyses, we only included studies with at least 3 levels of physical activity exposure, a requirement that limited the number of eligible results compared with previous meta-analyses.^7,8^ Physical activity exposures in primary studies were classified using a variety of methods and expressed in different formats, and this could have resulted in excluding even more studies. A key strength of the present meta-analytical work is our extensive exposure harmonization, maximizing the inclusion of the existing evidence. We did observe high heterogeneity between study estimates, but differences in method of exposure harmonization did not explain variation in effect sizes. Duration of follow-up, proportions of men and women, original analytical choices relating to the handling of other morbidities or prevalent depression at baseline, and method of depression diagnosis were not statistically significant predictors of effect size.

It is still possible that the associations observed in the present analyses could overestimate the role of physical activity because of reverse causality. That is, depression at baseline results in lower physical activity in participants who then go on to record depression during follow-up. For depression diagnosed at baseline, this bias is often mitigated by excluding these prevalent cases from analyses. Two of the studies included in our meta-analysis did not exclude participants with depression at baseline or adjust for these statistically,^24,33^ and our subgroup analysis indicated (although not statistically significantly so) that effect sizes of this subgroup were stronger than those from studies that excluded prevalent cases. Reverse causality bias may also result from depression that is undiagnosed at baseline, particularly given the often recurring nature of the condition.^52^ To limit risk of bias from undiagnosed depression, we only included studies with at least 3 years of follow-up. This does not rule out the possibility that physical activity may be causally associated with depression on a shorter time scale. Two studies included in our full-text screening were excluded solely based on follow-up time. A study of 108 000 South Korean adults reported an inverse association for physical activity in the exposure range approximating 7 to 40 mMET hours per week, but no association at very high activity levels.^53^ A study of 4600 Irish adults reported lower (albeit not statistically significant) odds of depression in those meeting physical activity guidelines compared with those who did not.^54^

Longer follow-up time limits the risk of reverse causality affecting the results but can also introduce regression dilution bias owing to exposure measurement error caused by true variation in physical activity behavior during follow-up.^55^ For example, in a recent study, mortality associations estimated using 28 years’ follow-up with a single measure of physical activity at baseline were 2- to 3-fold weaker than those estimated using repeated measures.^56^ We did not include results using more than 1 measure of physical activity because none were identified that met our inclusion criteria. However, we did observe weaker associations in studies with longer follow-up times, although again, this meta-regression result was not statistically significant. Given the recurrent nature of depression and engagement in physical activity as a treatment for this condition,^57^ use of repeated measures may be particularly important and would strengthen the evidence base for evaluating the dose-response relationship. Further work is required to understand the effects of different strategies for managing reverse causality and exposure measurement error in studies of depression.

### Limitations

A limitation of these analyses is that included studies only used self-reported measures of physical activity, which are subject to recall and social-desirability biases. Our analyses also included relatively limited data at higher physical activity doses. Studies using device-based measures of activity capturing a wider range of exposure and with longer follow-up of incident depression are therefore warranted. There were insufficient studies with stratified results to examine sex, age, or geographical subgroups, and notably, lower- and middle-income countries were underrepresented in included studies.

## Conclusions

This meta-analysis found an association between physical activity and incident depression. This suggests substantial mental health benefits can be achieved at physical activity levels even below the public health recommendations, with additional benefit for meeting the minimum recommended target but limited extra benefit beyond that. Assuming causality, 1 in 9 cases of depression might have been prevented if everybody in the population was active at the level of current health recommendations.

## Notes

Supplement. **eMethods 1.** Search strings **eMethods 2.** Data imputation procedures **eMethods 3.** Estimating the resting component of energy expenditure **eTable 1.** Study inclusion and exclusion criteria **eTable 2.** Study characteristics for analysis of heterogeneity **eTable 3.** Relative risks using alternate assumptions **eTable 4.** Potential impact fractions using alternate assumptions **eFigure 1.** Study screening and selection flowchart **eFigure 2.** Distribution of marginal MET hours per week **eFigure 3.** Subgroup analysis **eFigure 4.** Leave-one-out sensitivity analysis
eReferences
