# Supplementary material for: Association Between Physical Activity and Risk of Depression: A Systematic Review and Meta-analysis
Source: JAMA Psychiatry. 2022 Apr 13;79(6):550–9. doi: 10.1001/jamapsychiatry.2022.0609 (PMC9008579; doi:10.1001/jamapsychiatry.2022.0609)
Supplement: Supplement. — eMethods 1. Search strings eMethods 2. Data imputation procedures eMethods 3. Estimating the resting component of energy expenditure eTable 1. Study inclusion and exclusion criteria eTable 2. Study characteristics for analysis of heterogeneity eTable 3. Relative risks using alternate assumptions eTable 4. Potential impact fractions using alternate assumptions eFigure 1. Study screening and selection flowchart eFigure 2. Distribution of marginal MET hours per week eFigure 3. Subgroup analysis eFigure 4. Leave-one-out sensitivity analysis eReferences [file jamapsychiatry-e220609-s001.pdf]

Supplemental Online Content

Pearce M, Garcia L, Abbas A, et al. Association between physical activity and risk of depression: a systematic review and meta-analysis. *JAMA Psychiatry*. Published online April 13, 2022. doi:10.1001/jamapsychiatry.2022.0609

eMethods 1. Search strings 2

eMethods 2. Data imputation procedures 5

eMethods 3. Estimating the resting component of energy expenditure 8

eTable 1. Study inclusion and exclusion criteria 9

eTable 2. Study characteristics for analysis of heterogeneity 10

eTable 3. Relative risks using alternate assumptions 13

eTable 4. Potential impact fractions using alternate assumptions 14

eFigure 1. Study screening and selection flowchart 15

eFigure 2. Distribution of marginal MET hours per week 16

eFigure 3. Subgroup analysis 17

eFigure 4. Leave-one-out sensitivity analysis 18

eReferences 19

This supplemental material has been provided by the authors to give readers additional information about their work.

© 2022 Pearce M et al. *JAMA Psychiatry*.

eMethods 1: Search strings

As stated in PROSPERO record CRD42018095507, the searches included terms for dementia and Parkinson's disease for use in a separate meta-analysis, with papers filtered accordingly at the full-text screening stage. See Supplementary Figure 1 for further details.

Search string for Pubmed

((physical activit*[Title/Abstract]) OR (physical inactivity[Title/Abstract]) OR (exercise[Title/Abstract]) OR (exercises[Title/Abstract]) OR (exercising[Title/Abstract]) OR (recreational activit*[Title/Abstract]) OR (sport[Title/Abstract]) OR (sports[Title/Abstract]) OR (active transport[Title/Abstract]) OR (active transportation[Title/Abstract]) OR (active commut*[Title/Abstract]) OR (active travel*[Title/Abstract]) OR (household activit*[Title/Abstract]) OR (housework[Title/Abstract]) OR (non-exercise activit*[Title/Abstract]) OR (nonexercise activit*[Title/Abstract]) OR (activities of daily living[Title/Abstract]) OR (bicycle[Title/Abstract]) OR (bicycling[Title/Abstract]) OR (bike[Title/Abstract]) OR (biking[Title/Abstract]) OR (walk[Title/Abstract]) OR (walking[Title/Abstract]) OR (run[Title/Abstract]) OR (running[Title/Abstract]) OR (jogging[Title/Abstract]) OR (swim[Title/Abstract]) OR (swimming[Title/Abstract]) OR (resistance training[Title/Abstract]) OR (weight lifting[Title/Abstract]) OR (physical fitness[Title/Abstract]) OR (physical endurance[Title/Abstract]) OR (activity energy expenditure[Title/Abstract]) OR (caloric expenditure[Title/Abstract]) OR (motor activity[Mesh:NoExp]) OR (exercise[Mesh:NoExp]) OR (human physical conditioning[Mesh]) OR (leisure activities[Mesh:NoExp]) OR (recreation[Mesh:NoExp]) OR (dancing[Mesh]) OR (gardening[Mesh]) OR (sports[Mesh]) OR (activities of daily living[Mesh:NoExp]))

AND

((depression[title/abstract]) OR (depressive[title/abstract]) OR (dementia[Title/Abstract]) OR (Alzheimer[Title/Abstract]) OR (Alzheimer's[Title/Abstract]) OR (Parkinson[Title/Abstract]) OR (Parkinson's[Title/Abstract]) OR (depression[Mesh]) OR (depressive disorder[Mesh:NoExp]) OR (major depressive disorder[Mesh]) OR (dysthymic disorder[Mesh]) OR (dementia[MeSH:NoExp]) OR (Alzheimer disease[MeSH]) OR (vascular dementia[MeSH]) OR (Parkinson disease[Mesh]) OR (secondary Parkinson disease[Mesh]))

AND

((cohort[Title/Abstract]) OR (cohorts[Title/Abstract]) OR (follow-up study[Title/Abstract]) OR (follow-up studies[Title/Abstract]) OR (prospective study[Title/Abstract]) OR (prospective studies[Title/Abstract]) OR (longitudinal study[Title/Abstract]) OR (longitudinal studies[Title/Abstract]) OR (cohort studies[Mesh:NoExp]) OR (follow-up studies[Mesh]) OR (longitudinal studies[Mesh:NoExp]) OR (prospective studies[Mesh]))

Search string for SCOPUS

(TITLE-ABS-KEY("physical activit*") OR TITLE-ABS-KEY("physical inactivity") OR TITLE-ABS-KEY(exercise) OR TITLE-ABS-KEY(exercises) OR TITLE-ABS-KEY(exercising) OR TITLE-ABS-KEY("recreational activit*") OR TITLE-ABS-KEY(sport) OR TITLE-ABS-KEY(sports) OR TITLE-ABS-KEY("active transport") OR TITLE-ABS-KEY("active transportation") OR TITLE-ABS-KEY("active commut*") OR TITLE-ABS-KEY("active travel*") OR TITLE-ABS-KEY("household activit*") OR TITLE-ABS-KEY(housework) OR TITLE-ABS-KEY("non-exercise activit*") OR TITLE-ABS-KEY("nonexercise activit*") OR TITLE-ABS-KEY("activities of daily living") OR TITLE-ABS-KEY(bicycle) OR TITLE-ABS-KEY(bicycling) OR TITLE-ABS-KEY(bike) OR TITLE-ABS-KEY(biking) OR TITLE-ABS-KEY(walk) OR TITLE-ABS-KEY(walking) OR TITLE-ABS-KEY(run) OR TITLE-ABS-KEY(running) OR TITLE-ABS-KEY(jogging) OR TITLE-ABS-KEY(swim) OR TITLE-ABS-KEY(swimming) OR TITLE-ABS-KEY("resistance training") OR TITLE-ABS-KEY("weight lifting") OR TITLE-ABS-KEY("physical fitness") OR TITLE-ABS-KEY("physical endurance") OR TITLE-ABS-KEY("activity energy expenditure") OR TITLE-ABS-KEY("caloric expenditure"))

AND

© 2022 Pearce M et al. *JAMA Psychiatry*.

(TITLE-ABS-KEY(depression) OR TITLE-ABS-KEY(depressive) OR TITLE-ABS-KEY(dementia) OR TITLE-ABS-KEY(Alzheimer) OR TITLE-ABS-KEY(Alzheimer’s) OR TITLE-ABS-KEY(Parkinson) OR TITLE-ABS-KEY(Parkinson’s))

AND

(TITLE-ABS-KEY(cohort) OR TITLE-ABS-KEY(cohorts) OR TITLE-ABS-KEY(“follow-up study”) OR TITLE-ABS-KEY(“follow-up studies”) OR TITLE-ABS-KEY(“prospective study”) OR TITLE-ABS-KEY(“prospective studies”) OR TITLE-ABS-KEY(“longitudinal study”) OR TITLE-ABS-KEY(“longitudinal studies”))

Search string for Web of Science

(TS=(“physical activit*”) OR TS=(“physical inactivity”) OR TS=(exercise) OR TS=(exercises) OR TS=(exercising) OR TS=(“recreational activit*”) OR TS=(sport) OR TS=(sports) OR TS=(“active transport”) OR TS=(“active transportation”) OR TS=(“active commut*”) OR TS=(“active travel*”) OR TS=(“household activit*”) OR TS=(housework) OR TS=(“non-exercise activit*”) OR TS=(“nonexercise activit*”) OR TS=(“activities of daily living”) OR TS=(bicycle) OR TS=(bicycling) OR TS=(bike) OR TS=(biking) OR TS=(walk) OR TS=(walking) OR TS=(run) OR TS=(running) OR TS=(jogging) OR TS=(swim) OR TS=(swimming) OR TS=(“resistance training”) OR TS=(“weight lifting”) OR TS=(“physical fitness”) OR TS=(“physical endurance”) OR TS=(“activity energy expenditure”) OR TS=(“caloric expenditure”))

AND

(TS=(depression) OR TS=(depressive) OR TS=(dementia) OR TS=(Alzheimer) OR TS=(Alzheimer’s) OR TS=(Parkinson) OR TS=(Parkinson’s))

AND

(TS=(cohort) OR TS=(cohorts) OR TS=(“follow-up study”) OR TS=(“follow-up studies”) OR TS=(“prospective study”) OR TS=(“prospective studies”) OR TS=(“longitudinal study”) OR TS=(“longitudinal studies”))

Search string for PsycINFO

((Keywords: “physical activit*” OR “physical inactivity” OR exercise OR exercises OR exercising OR “recreational activit*” OR sport OR sports OR “active transport” OR “active transportation” OR “active commut*” OR “active travel*” OR “household activit*” OR housework OR “non-exercise activit*” OR “nonexercise activit*” OR “activities of daily living” OR bicycle OR bicycling OR bike OR biking OR walk OR walking OR run OR running OR jogging OR swim OR swimming OR “resistance training” OR “weight lifting” OR “physical fitness” OR “physical endurance” OR “activity energy expenditure” OR “caloric expenditure”) OR (Abstract: “physical activit*” OR “physical inactivity” OR exercise OR exercises OR exercising OR “recreational activit*” OR sport OR sports OR “active transport” OR “active transportation” OR “active commut*” OR “active travel*” OR “household activit*” OR housework OR “non-exercise activit*” OR “nonexercise activit*” OR “activities of daily living” OR bicycle OR bicycling OR bike OR biking OR walk OR walking OR run OR running OR jogging OR swim OR swimming OR “resistance training” OR “weight lifting” OR “physical fitness” OR “physical endurance” OR “activity energy expenditure” OR “caloric expenditure”))

AND

((Keywords: depression OR depressive OR dementia OR Alzheimer OR Alzheimer’s OR Parkinson OR Parkinson’s) OR (Abstract: depression OR depressive OR dementia OR Alzheimer OR Alzheimer’s OR Parkinson OR Parkinson’s))

AND

((Keywords: cohort OR cohorts OR “follow-up study” OR “follow-up studies” OR “prospective study” OR “prospective studies” OR “longitudinal study” OR “longitudinal studies”) OR (Abstract: cohort OR

© 2022 Pearce M et al. *JAMA Psychiatry*.

cohorts OR “follow-up study” OR “follow-up studies” OR “prospective study” OR “prospective studies” OR “longitudinal study” OR “longitudinal studies”))

© 2022 Pearce M et al. *JAMA Psychiatry*.

eMethods 2: Data imputation procedures

CRITICAL INFORMATION (required to run the meta-analysis)

1) Number of individuals in each exposure category (required only when risk estimate presented as relative risk or odds ratio):

I. Authors contacted

II. Or estimated via proportional weighting using the following equation:

$$\frac{p_i}{P}S \tag{1}$$

Where p_i is person-years in the ith exposure category, P is total person-years, and S is total analytical sample size.

III. Or estimated via proportional weighting using the following steps (when person-years in each exposure category was not reported):

$$w_i = \frac{c_i}{(c_1 RR_i)} \tag{3}$$

$$s_1 = \frac{S}{\sum_{i=1}^{n} w_i} \tag{4}$$

$$s_i = w_i s_1 \tag{5}$$

Where w_i is the ratio between person-years in the ith exposure category and in the reference exposure category, c_i is cases in the ith exposure category (subscript 1 refers to the reference level), RR_i is minimally adjusted relative risk in the ith exposure category, s_i is number of individuals in the ith exposure category, and S is total analytical sample size.

Equations 3 and 5 derive from:

$$RR_i = \frac{\frac{c_i}{s_i}}{\frac{c_1}{s_1}} = \frac{c_i s_1}{c_1 s_i} \rightarrow s_i = \frac{c_i s_1}{c_1 RR_i} \rightarrow \frac{s_i}{s_1} = \frac{c_i}{c_1 RR_i} \tag{6}$$

Equation 4 derives from:

$$S = \sum_{i=1}^{n} s_i = \sum_{i=1}^{n} w_i s_1 \tag{7}$$

Once s_1 is found, it can be used in Equation 5 to estimate number of individuals in all other exposure categories.

2) Person-years in each exposure category (required only when risk estimate presented as hazard ratio):

I. Authors contacted

II. Or estimated via proportional weighting using the following equation:

© 2022 Pearce M et al. *JAMA Psychiatry*.

$$\frac{n_i}{S}P \tag{2}$$

Where n_i is number of individuals in the ith exposure category, S is total analytical sample size, and P is total person-years.

III. Or estimated via proportional weighting (when number of individuals in each exposure category was not reported) using Equations 3 to 5 substituting the number of individuals in the ith exposure category (s_i) and total analytical sample size (S) by person-years in the ith exposure category and total person-years, respectively.

3) Cases in each exposure category:

I. Authors contacted

II. Or estimated via proportional weighting using the following steps:

$$c_{1_t} = \frac{RR_1}{\sum_{i=1}^{n} RR_i} C \tag{8}$$

$$r_1 = \frac{c_{1_t}}{n_1} \tag{9}$$

$$r_i = RR_i r_1 \tag{10}$$

$$c_i = n_i r_i \frac{C}{\sum_{i=1}^{n} (n_i r_i)} \tag{11}$$

Where c_i is cases in the ith exposure category (subscript 1 refers to the reference level; subscript t indicates temporary (non-corrected) number of cases), RR_i is risk estimate in the ith exposure category, C is total number of cases, r_i is risk in the ith exposure category, n_i is person-years (or number of individuals) in the ith exposure category. Equation 11 is also used to estimate the final number of cases in the reference level.

BACKGROUND INFORMATION (obtained or estimated only when needed to impute at least one of the critical information)

1) Total analytical sample size:

I. Calculated via summation of the number of individuals in each exposure category.

II. Or reported in the paper.

III. Or authors contacted.

IV. Or estimated dividing total person-years by mean follow-up.

2) Mean follow-up:

I. Reported in the paper (mean or median follow-up).

II. Or authors contacted.

© 2022 Pearce M et al. *JAMA Psychiatry*.

III. Or calculated by dividing total person-years by total analytical sample size.
IV. Or reported study follow-up duration (e.g., 1990 to 2010 = 20 years), as an approximate surrogate.

3) Total person-years:

I. Calculated via summation of person-years in each exposure category.
II. Or reported in the paper.
III. Or authors contacted.
IV. Or estimated multiplying total analytical sample size by mean follow-up.

4) Total number of cases:

I. Calculated via summation of cases in each exposure category.
II. Or reported in the paper.
III. Or authors contacted.

© 2022 Pearce M et al. *JAMA Psychiatry*.

eMethods 3: Estimating the resting component of energy expenditure to convert METs to mMETs.

Total physical activity volume can be calculated by multiplying duration by the rate of energy expenditure (intensity). Rate of energy expenditure is often expressed in gross units of metabolic equivalent of task or MET which includes both a resting (1 MET) and an activity component (the remainder, e.g. 3.5 METs for a 4.5 gross MET activity).

When publications report activity volume in total MET.hrs (hours*METs) per week, it is necessary to obtain information on the duration of activities making up this total such that 1 MET can be subtracted for each hour to remove the resting component and give physical activity energy expenditure in marginalised METs (mMETs).

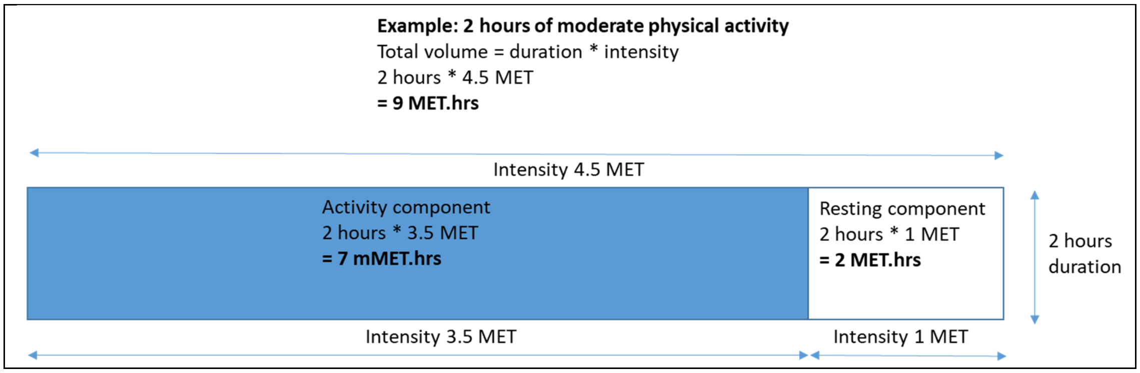

In the above example, a study reports total physical activity volume as 9 MET.hrs. Knowledge of the duration of activity of 2 hours is necessary to calculate and remove the resting component giving marginalised METs (mMETs).

For studies that did not provide activity duration, a conversion equation from gross to marginal units is required. For the current analyses, we used a regression equation derived from the studies that provided both estimates, weighted for N per exposure category:

mMET.hrs = 0.89 MET.hrs -0.52 $\text{MET.hrs}^{0.5}$

The studies/cohorts contributing to this equation were identified in a separate systematic review and meta-analysis (see PROSPERO, registration number CRD42018095481):

- Lee et al. (2011), Aerobics Center Longitudinal Study[1]
- Chomistek et al. (2012), Health Professionals Follow-up Study[2]
- Chomistek et al. (2013), Women's Genome Health Study[3]
- Wen et al. (2011), Taiwan Medical Screening Program[4]
- Mok et al. (2012), Severance Cohort Study[5]
- Hu et al. (2000), Nurses' Health Study[6]
- Besson et al, EPIC-Norfolk[7]

© 2022 Pearce M et al. *JAMA Psychiatry*.

eTable 1 Study inclusion and exclusion criteria.

Include	Exclude
Participants	
Human adults (18+ years of age)	Cohorts of institutionalised adults, medical patients (i.e., in secondary or tertiary prevention) or high-risk populations (e.g., hypertension, diabetes - except conditions inevitable with ageing, as post-menopause)
Interventions	
Volume (marginal MET-hrs/week) of non-occupational physical activity – this includes leisure-time physical activity alone, combined with other non-occupational physical activity domains (e.g., transport and/or domestic activity), or combined with specific types of activity such as walking. Physical activity volume can be either self-reported or measured by devices and must be reported in at least three exposure levels	Measures of physical activity from which occupational activity cannot be factored out; individual domains of physical activity which occur outside of work but do not include leisure-time activity (e.g., travel physical activity only); other risk factors either alone or combined with physical activity
Comparators	
Lower volumes of physical activity (as defined in 'Interventions')	
Outcomes	
Depression (diagnosed by a physician, hospital discharge register or any validated scale with a cut-off for depressive symptoms) (F32-F33)*	Less than three years of follow-up period
Study design	
General population prospective cohort studies (including case-cohort and nested case-control studies) with sample size larger than 3,000 participants; results must be reported either as hazard ratios, relative risks or odds ratios with confidence intervals	Other study designs (e.g., experimental or cross-sectional), even when nested in prospective cohort studies
* Diseases coded using the 10th Revision of International Statistical Classification of Diseases, version 2018.	

- ✓ Type of documents: peer-reviewed papers published in academic journals.
- ✓ No limits on language and date of publication.

© 2022 Pearce M et al. *JAMA Psychiatry*.

eTable 2 Study characteristics for analysis of heterogeneity.

First author	Outcome	Outcome ascertainment	Exposure harmonisation category	Follow-up years (above or below median)	Exclusion of cases at baseline	Other morbidities	Exclusion of new cases at start of follow-up	Covariates
Mikkelsen, S.[8]	Major depression	Registry	Frequency/duration/intensity assumptions	≥ 8.5 years	Yes	Statistical adjustment	No	Income, education, smoking, alcohol intake, body mass index, occupational activity, chronic disease
Paffenbarger, Jr R.[9]	Major depression	Self-report of physician diagnosis	Measurement unit conversion followed by resting energy expenditure equation	≥ 8.5 years	No	Exclusion at baseline	No	Age
Ten Have, M.[10]	Major depression	Electronic CIDI[11]	Frequency/duration/intensity assumptions	< 8.5 years	Yes	Statistical adjustment	No	Gender, age, education, partner status, employment status, presence of a somatic illness
Chang, S.[12]	Major depression	Self-report of depressive symptoms, use of antidepressants, physician diagnosis,	Frequency/duration/intensity assumptions	< 8.5 years	Yes	Statistical adjustment	24 months	Age, education level, race/ethnicity, social network, body mass index, alternate Mediterranean diet score, cigarette smoking, largest number of drinks in a single day, medical comorbidity, hours of actual sleep per day, physical/functional limitation
Cabello, M.[13]	Major depression	World Mental Health Survey Initiative version of the (CIDI)[14], self-report of physician diagnosis, medication, or treatment	Measurement unit conversion followed by resting energy expenditure equation plus occupational physical activity assumption	< 8.5 years	Yes	Statistical adjustment	No	Demographics, presence of a physical chronic condition, body mass index, general health status, country
Fernandez-Montero, A.[15]	Major depression	Self-report of physician diagnosis	Measurement unit conversion followed by resting energy expenditure equation	< 8.5 years	Yes	Exclusion at baseline	No	Sex, baseline body mass index, time sleeping, time nap, time TV, total energy intake, adherence to the Mediterranean Diet, alcohol intake, smoking pack years, educational level, hypertension, diabetes mellitus, cancer, changes in physical activity in the 2th and

© 2022 Pearce M et al. *JAMA Psychiatry*.

First author	Outcome	Outcome ascertainment	Exposure harmonisation category	Follow-up years (above or below median)	Exclusion of cases at baseline	Other morbidities	Exclusion of new cases at start of follow-up	Covariates
								4th year follow-up, with age and year of entering the cohort as stratification variables
Hallgren, M.[16]	Major depression	Registry	Frequency/duration/intensity assumptions	≥ 8.5 years	Yes	Statistical adjustment	No	Sex, age, occupation, smoking, body mass index, comorbidities
Espana-Romero, V.[17]	Elevated depressive symptoms	10-item CES-D scale[18]	Measurement unit conversion followed by resting energy expenditure equation	≥ 8.5 years	Yes	Exclusion at baseline	No	Age, sex, baseline examination year, survey response year, alcohol consumption, smoking, BMI, diet, total cholesterol, blood pressure, blood glucose
Camacho, T.[19]	Elevated depressive symptoms	Human Population Laboratory depression index[20]	Frequency/duration/intensity assumptions	≥ 8.5 years	Yes	Statistical adjustment	No	Age, physical health, socioeconomic status, social supports, life events, anomy, alcohol consumption, smoking status, relative weight
Pavey, T.[21]	Elevated depressive symptoms	10-item CES-D scale[18]	Measurement unit conversion followed by resting energy expenditure equation	< 8.5 years	No	Statistical adjustment	No	Age, education, marital status, area of residence, smoking, alcohol, sitting, chronic conditions, body mass index
Wise, L.[22]	Elevated depressive symptoms	20-item CES-D scale[23]	Frequency/duration/intensity assumptions	< 8.5 years	Yes	Statistical adjustment	No	Age, education, occupation, marital status, geographic region, non-vigorous physical activity body mass index, pre-existing health conditions, energy intake, smoking, current alcohol consumption, child care responsibilities
Hamer, M.[24]	Elevated depressive symptoms	8-item CES-D scale[25]	Frequency/duration/intensity assumptions	< 8.5 years	Yes	Statistical adjustment	No	Age, gender, baseline CES-D score, social occupational class, smoking, alcohol, longstanding illness, C-reactive protein

© 2022 Pearce M et al. *JAMA Psychiatry*.

First author	Outcome	Outcome ascertainment	Exposure harmonisation category	Follow-up years (above or below median)	Exclusion of cases at baseline	Other morbidities	Exclusion of new cases at start of follow-up	Covariates
Kuwahara, K.[26]	Elevated depressive symptoms	13-item cohort specific scale[27]	Measurement unit conversion followed by resting energy expenditure equation	< 8.5 years	Yes	Exclusion at baseline	No	Age, sex, body mass index, smoking, alcohol consumption, shift work, overtime work, job position, marital status, mutual relations, baseline depression scores.
Harvey, S.[28]	Elevated depressive symptoms	14-item Hospital Anxiety and Depression scale[29]	Frequency/duration/intensity assumptions	≥ 8.5 years	Yes	Exclusion at baseline	No	Age, gender, marital status, education, social class, number of cigarettes consumed, alcohol use, body mass index
Hughes, K.[30]	Elevated depressive symptoms	5-item Mental Health Inventory[31]	Measurement unit conversion followed by resting energy expenditure equation	≥ 8.5 years	No	Exclusion at baseline	No	Age, caffeine intake, alcohol intake

CES-D = Center for Epidemiologic Studies Depression Scale; mMET = marginal metabolic equivalent of task; SD = standard deviation

© 2022 Pearce M et al. *JAMA Psychiatry*.

eTable 3 Relative risk of depression, major depression, and depressive symptoms at three physical activity levels (in mMET-hrs/wk), comparing main meta-analysis results with meta-analysis using alternative assumptions for physical activity intensity and session duration, and alternative placement of last knots at 0th, 42.5th, and 85th percentiles of person-years.

**Outcome**	**Main results**<br><br>**Activity volume (mMET-hrs/week)**				**Alternative PA intensity and duration assumptions**<br><br>**Activity volume (mMET-hrs/week)**				**Alternative knot placements (knots at 0th, 42.5th, 85th percentiles)**<br><br>**Activity volume (mMET-hrs/week)**		
	**4.4**	**8.8**	**17.5**		**4.4**	**8.8**	**17.5**		**4.4**	**8.8**	**17.5**
	**RR (95%CI)**	**RR (95%CI)**	**RR (95%CI)**		**RR (95%CI)**	**RR (95%CI)**	**RR (95%CI)**		**RR (95%CI)**	**RR (95%CI)**	**RR (95%CI)**
Depression	0.82 (0.77-0.87)	0.75 (0.68-0.82)	0.72 (0.64-0.81)		0.80 (0.74-0.86)	0.73 (0.66-0.82)	0.71 (0.62-0.81)		0.85 (0.81-0.89)	0.76 (0.70-0.83)	0.72 (0.64-0.81)
Major depression	0.83 (0.75-0.92)	0.75 (0.64-0.87)	0.74 (0.61-0.88)		0.80 (0.70-0.91)	0.73 (0.61-0.87)	0.73 (0.58-0.91)		0.85 (0.78-0.93)	0.77 (0.67-0.88)	0.74 (0.62-0.89)
Elevated depressive symptoms	0.80 (0.73-0.88)	0.73 (0.64-0.84)	0.70 (0.59-0.84)		0.78 (0.69-0.87)	0.73 (0.63-0.85)	0.70 (0.58-0.84)		0.83 (0.77-0.90)	0.74 (0.65-0.84)	0.69 (0.58-0.82)

mMET = marginal metabolic equivalent of task; RR =relative risk; 95%CI = 95% confidence interval. Relative accumulating 0 mMET-hrs/week.

© 2022 Pearce M et al. *JAMA Psychiatry*.

eTable 4 Potential impact fractions of physical activity on depression, major depression, and depressive symptoms at three physical activity levels (in mMET-hrs/wk), comparing main meta-analysis results with meta-analysis using alternative assumptions for physical activity intensity and session duration, and alternative placement of last knots at 0th, 42.5th, and 85th percentiles of person-years.

**Outcome**	**Main results**<br><br>**Activity volume (mMET-hrs/week)**			**Alternative PA intensity and duration assumptions**<br><br>**Activity volume (mMET-hrs/week)**			**Alternative knot placements (knots at 0th, 42.5th, 85th percentiles)**<br><br>**Activity volume (mMET-hrs/week)**		
	**4.4**	**8.8**	**17.5**	**4.4**	**8.8**	**17.5**	**4.4**	**8.8**	**17.5**
	**PIF (95%CI)**	**PIF (95%CI)**	**PIF (95%CI)**	**PIF (95%CI)**	**PIF (95%CI)**	**PIF (95%CI)**	**PIF (95%CI)**	**PIF (95%CI)**	**PIF (95%CI)**
Depression	6.38% (4.25-8.63)	11.53% (7.69-15.43)	13.89% (8.44-19.25)	7.74% (4.99-10.61)	12.37% (7.95-16.82)	14.33% (8.29-20.21)	5.50% (3.69-7.41)	11.10% (7.47-14.77)	14.85% (9.26-20.30)
Major depression	2.97% (1.27-4.91)	7.28% (3.36-11.44)	8.04% (2.38-13.82)	3.86% (1.53-6.53)	7.63% (3.23-12.35)	7.62% (0.14-15.13)	2.62% (1.11-4.34)	6.98% (3.21-11.00)	8.46% (2.75-14.27)
Depressive symptoms	9.45% (5.19-13.86)	14.44% (7.88-20.92)	17.01% (8.39-25.24)	11.15% (5.88-16.52)	14.77% (7.64-21.79)	17.60% (8.18-26.49)	8.09% (4.53-11.79)	14.66% (8.27-20.98)	19.96% (9.87-27.52)

mMET = marginal metabolic equivalent of task; PIF = potential impact fraction; 95%CI = 95% confidence interval. Relative accumulating 0 mMET-hrs/week.

© 2022 Pearce M et al. *JAMA Psychiatry*.

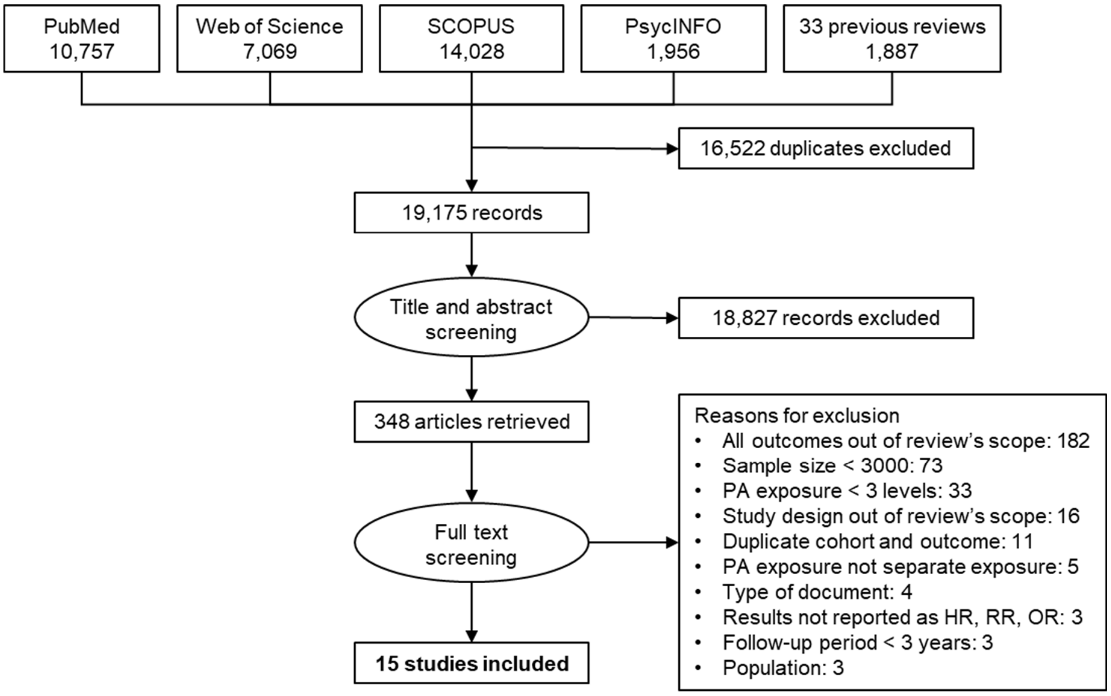

eFigure 1 Study screening and selection flowchart.

© 2022 Pearce M et al. *JAMA Psychiatry*.

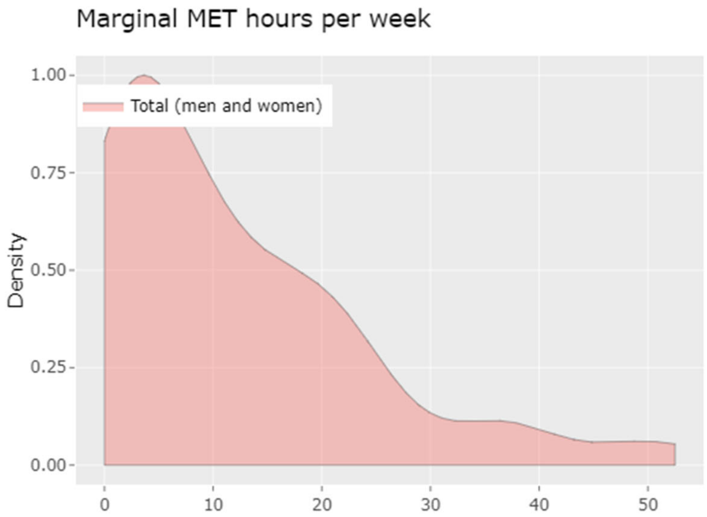

Depression

Physical activity (mMET-hrs/week)

Min: 0.0
25th percentile: 2.7
50th percentile: 8.4
Mean: 11.7
75th percentile: 18.0
Max: 53.0

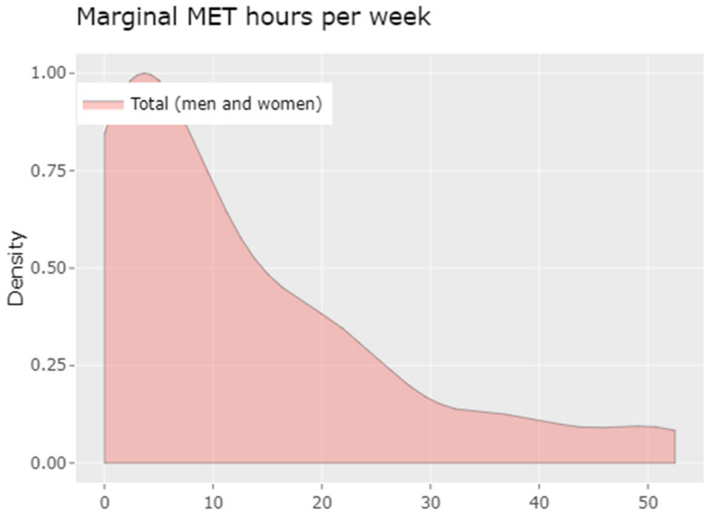

Elevated depressive symptoms

Physical activity (mMET-hrs/week)

Min: 0.0
25th percentile: 2.6
50th percentile: 7.9
Mean: 12.3
75th percentile: 17.8
Max: 53.0

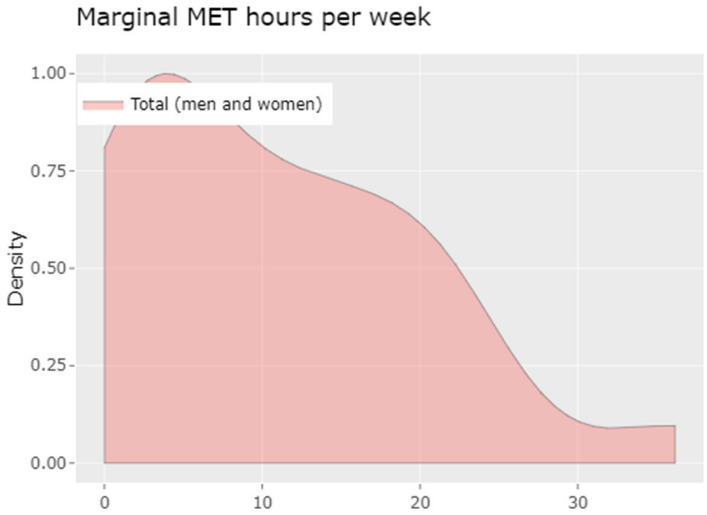

Major depression

Physical activity (mMET-hrs/week)

Min: 0.0
25th percentile: 3.8
50th percentile: 9.0
Mean: 10.7
75th percentile: 17.3
Max: 36.1

eFigure 2 Distribution of marginal MET hours per week (mMET-hrs/week) for cohorts included depression, depressive symptoms, and major depression analyses.

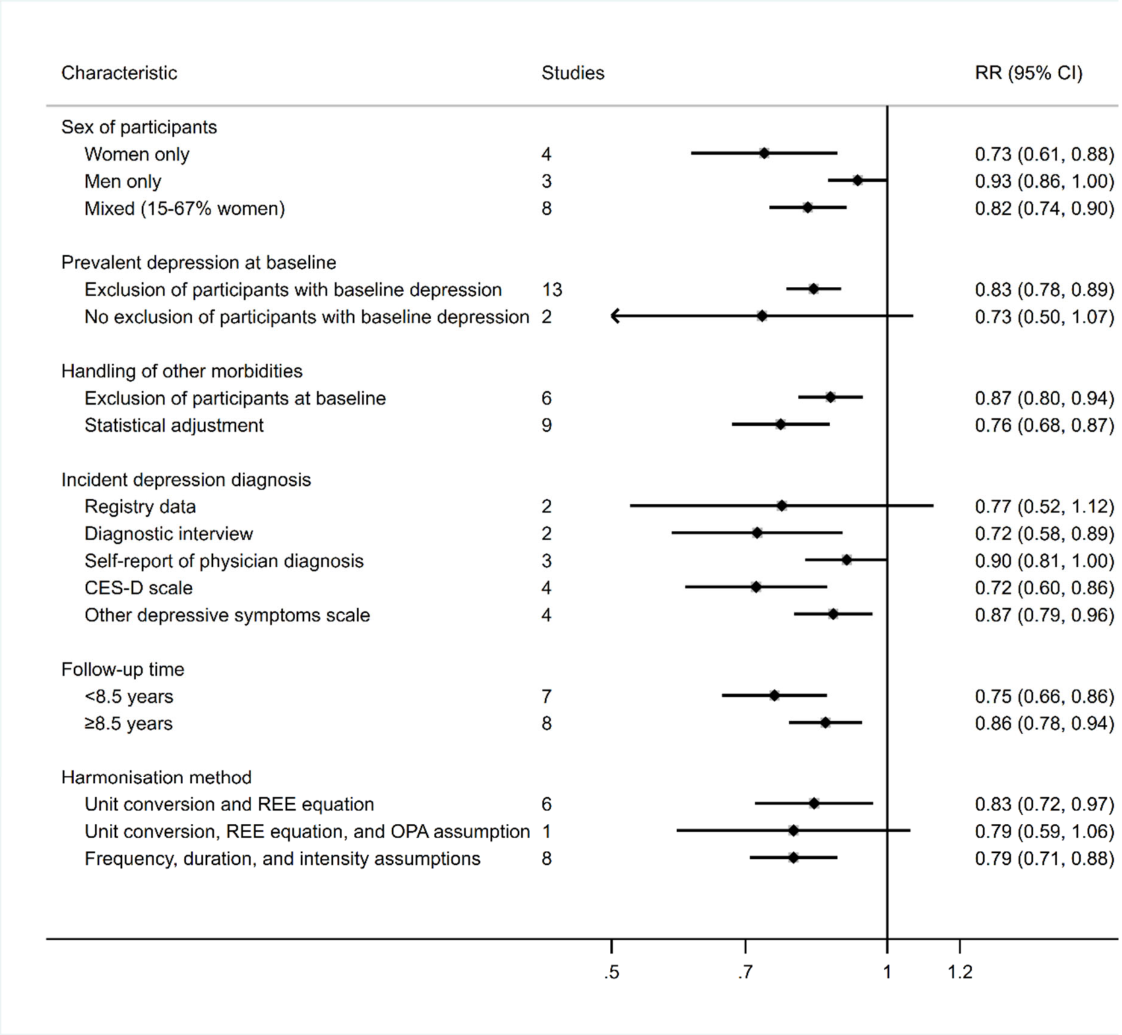

eFigure 3 Subgroup analysis of associations between physical activity (8.8 mMET-hrs/week vs 0 mMET-hrs/week) and incidence of major depression by study characteristics.
CES-D = Center for Epidemiologic Studies Depression Scale; CI = confidence interval; OPA = occupational physical activity; REE = resting energy expenditure; RR = relative risk.

Results of meta-regression for each study characteristic variable: sex of participants (R^2=15.6%; Wald chi^2=3.77, p=0.15), follow-up time (R^2=5.9%; Wald chi^2=2.11, p=0.15), harmonisation method (R^2=0.0%; Wald chi^2=0.34, p=0.84), prevalent depression at baseline (R^2=25.3%; Wald chi^2=2.01, p=0.16), handling of other morbidities (R^2=0.0%; Wald chi^2=1.77, p=0.18), incident depression diagnosis (R^2=23.4%; Wald chi^2=6.23, p=0.18).

© 2022 Pearce M et al. *JAMA Psychiatry*.

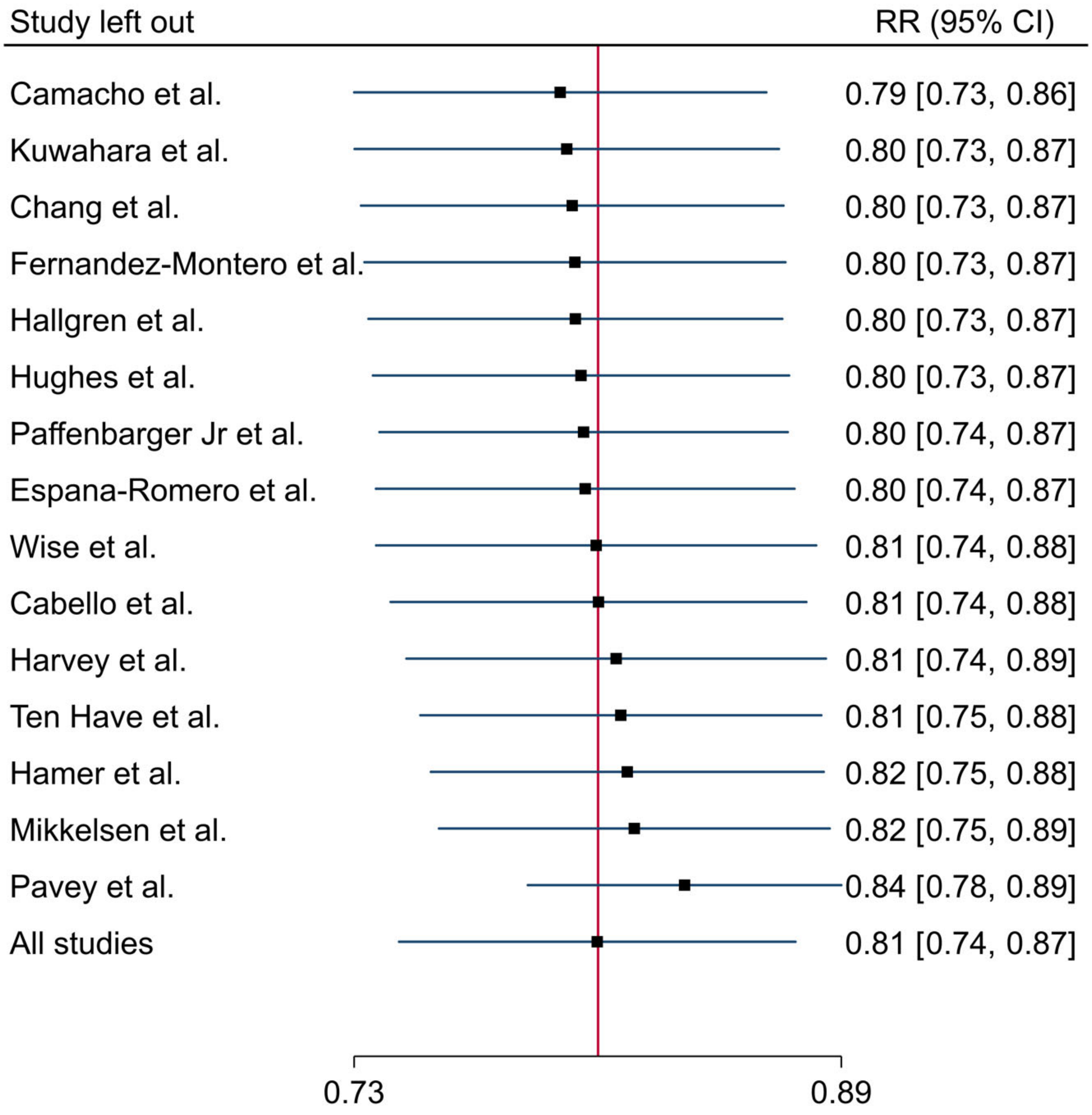

eFigure 4 Leave-one-out sensitivity analysis of associations between physical activity (8.8 mMET-hrs/week vs 0 mMET-hrs/week) and incidence of major depression. Vertical line indicates effect size in all 15 studies.
CI = confidence interval; RR = relative risk.

© 2022 Pearce M et al. *JAMA Psychiatry*.

eReferences

1. Lee DC, Sui X, Ortega FB, et al. Comparisons of leisure-time physical activity and cardiorespiratory fitness as predictors of all-cause mortality in men and women. *Br J Sports Med* 2011;

2. Chomistek AK, Cook NR, Flint AJ, et al. Vigorous-intensity leisure-time physical activity and risk of major chronic disease in men. *Med Sci Sports Exerc* 2012;44(10):1898–905.

3. Chomistek AK, Chasman DI, Cook NR, et al. Physical activity, genes for physical fitness, and risk of coronary heart disease. *Med Sci Sports Exerc* 2013;45(4):691–7.

4. Wen CP, Wai JPM, Tsai MK, et al. Minimum amount of physical activity for reduced mortality and extended life expectancy: A prospective cohort study. *Lancet* 2011;378(9798):1244–53. http://dx.doi.org/10.1016/S0140-6736(11)60749-6

5. Mok Y, Jeon C, Lee GJ, et al. Physical Activity Level and Colorectal Cancer Mortality. *Asia-Pacific J Public Heal* 2016;28(7):638–47.

6. Hu FB, Stampfer MJ, Colditz GA, et al. Physical activity and risk of stroke in women. *J Am Med Assoc* 2000;283(22):2961–7.

7. Besson H, Ekelund U, Brage S, et al. Relationship between subdomains of total physical activity and mortality. *Med Sci Sports Exerc* 2008;40(11):1909–15.

8. Mikkelsen SS, Tolstrup JS, Flachs EM, et al. A cohort study of leisure time physical activity and depression. *Prev Med (Baltim)* 2010;51(6):471–5. http://dx.doi.org/10.1016/j.ypmed.2010.09.008

9. Paffenbarger RS, Lee I -M, Leung R. Physical activity and personal characteristics associated with depression and suicide in American college men. *Acta Psychiatr Scand* 1994;

10. Ten Have M, de Graaf R, Monshouwer K. Physical exercise in adults and mental health status. Findings from the Netherlands Mental Health Survey and Incidence Study (NEMESIS). *J Psychosom Res* 2011;71(5):342–8. http://dx.doi.org/10.1016/j.jpsychores.2011.04.001

11. Smeets RM., Dingemans PMA. Composite International Diagnostic Interview (CIDI), Version 1.1. Geneva: World Health Organization; 1993.

12. Chang SC, Pan A, Kawachi I, et al. Risk factors for late-life depression: A prospective cohort study among older women. *Prev Med (Baltim)* 2016;

13. Cabello M, Miret M, Caballero FF, et al. The role of unhealthy lifestyles in the incidence and persistence of depression: A longitudinal general population study in four emerging countries. *Global Health* 2017;13(1):1–8.

14. Kessler RC, Üstün BB. The World Mental Health (WMH) Survey Initiative version of the World Health Organization (WHO) Composite International Diagnostic Interview (CIDI). International Journal of Methods in Psychiatric Research. 2004.

15. Fernandez-Montero A, Moreno-Galarraga L, Sánchez-Villegas A, et al. Erratum: Dimensions of leisure-time physical activity and risk of depression in the "seguimiento Universidad de Navarra" (SUN) prospective cohort (BMC Psychiatry (2020) 20 (98) DOI: 10.1186/s12888-020-02502-6). *BMC Psychiatry* 2020;20(1):1–9.

16. Hallgren M, Nguyen TTD, Lundin A, et al. Prospective associations between physical activity and clinician diagnosed major depressive disorder in adults: A 13-year cohort study. *Prev Med (Baltim)* 2019;118(October 2018):38–43. https://doi.org/10.1016/j.ypmed.2018.10.009

17. España-Romero V, Artero EG, Lee D chul, et al. A Prospective Study of Ideal Cardiovascular Health and Depressive Symptoms. *Psychosomatics* 2013;54(6):525–35. http://dx.doi.org/10.1016/j.psym.2013.06.016

18. Andersen ME, Carter BW, Malmgren AJ, et al. Screening for depression in well older adults: evaluation of a short form of the CES-D. *Am J Prev Med* 1994;10(2).

© 2022 Pearce M et al. *JAMA Psychiatry*.

19. Camacho TC, Roberts RE, Lazarus NB, et al. Physical activity and depression: Evidence from the alameda county study. *Am J Epidemiol* 1991;134(2):220–31.

20. KAPLAN GA, ROBERTS RE, CAMACHO TC, et al. Psychosocial Predictors of Depression. *Am J Epidemiol* 1987;125(2):206–20.

21. Pavey TG, Peeters G, Bauman AE, et al. Does vigorous physical activity provide additional benefits beyond those of moderate? *Med Sci Sports Exerc* 2013;45(10):1948–55.

22. Wise LA, Adams-Campbell LL, Palmer JR, et al. Leisure time physical activity in relation to depressive symptoms in the Black Women's Health Study. *Ann Behav Med* 2006;

23. Radloff LS. The CES-D Scale: A Self-Report Depression Scale for Research in the General Population. *Appl Psychol Meas* 1977;1(3):385–401.

24. Hamer M, Molloy GJ, de Oliveira C, et al. Leisure time physical activity, risk of depressive symptoms, and inflammatory mediators: The English Longitudinal Study of Ageing. *Psychoneuroendocrinology* 2009;34(7):1050–5.

25. Karim J, Weisz R, Bibi Z, et al. Validation of the Eight-Item Center for Epidemiologic Studies Depression Scale (CES-D) Among Older Adults. *Curr Psychol* 2015;34(4):681–92.

26. Kuwahara K, Honda T, Nakagawa T, et al. Associations of leisure-time, occupational, and commuting physical activity with risk of depressive symptoms among Japanese workers: A cohort study. *Int J Behav Nutr Phys Act* 2015;12(1):1–8. http://dx.doi.org/10.1186/s12966-015-0283-4

27. Yakura N. Verification of the Validity of Depressive Symptom Scale Based on the Existing Health Questionnaire. *Asian Pacific J Dis Manag* 2009;3(1):21–6.

28. Harvey SB, Overland S, Hatch SL, et al. Exercise and the prevention of depression: Results of the HUNT cohort study. *Am J Psychiatry* 2018;175(1):28–36.

29. Zigmond AS, Snaith RP. The Hospital Anxiety and Depression Scale. *Acta psychiatr scand* 1983;67:361–70.

30. Hughes KC, Gao X, Molsberry S, et al. Physical activity and prodromal features of Parkinson disease. *Neurology* 2019;93(23):E2157–69.

31. Berwick DM, Murphy JM, Goldman PA, et al. Performance of a five-item mental health screening test. *Med Care* 1991;29(2):169–76.

© 2022 Pearce M et al. *JAMA Psychiatry*.
